# A comparison of machine learning classifiers for smartphone-based gait analysis

**DOI:** 10.1007/s11517-020-02295-6

**Published:** 2021-02-06

**Authors:** Rosa Altilio, Andrea Rossetti, Qiang Fang, Xudong Gu, Massimo Panella

**Affiliations:** 1 Department of Information Engineering, Electronics and Telecommunications (DIET), University of Rome “La Sapienza”, Via Eudossiana 18, 00184 Rome, Italy; 2 Department of Biomedical Engineering, College of Engineering, Shantou University, Shantou, 515063 China; 3 Second Hospital of Jiaxing, Jiaxing, 314000 China

**Keywords:** Gait analysis, Machine learning classifier, Smartphone technology, Wavelet-based feature extraction, Home-based telemedicine

## Abstract

This paper proposes a reliable monitoring scheme that can assist medical specialists in watching over the patient’s condition. Although several technologies are traditionally used to acquire motion data of patients, the high costs as well as the large spaces they require make them difficult to be applied in a home context for rehabilitation. A reliable patient monitoring technique, which can automatically record and classify patient movements, is mandatory for a telemedicine protocol. In this paper, a comparison of several state-of-the-art machine learning classifiers is proposed, where stride data are collected by using a smartphone. The main goal is to identify a robust methodology able to assure a suited classification of gait movements, in order to allow the monitoring of patients in time as well as to discriminate among a pathological and physiological gait. Additionally, the advantages of smartphones of being compact, cost-effective and relatively easy to operate make these devices particularly suited for home-based rehabilitation programs.

**Figure:**
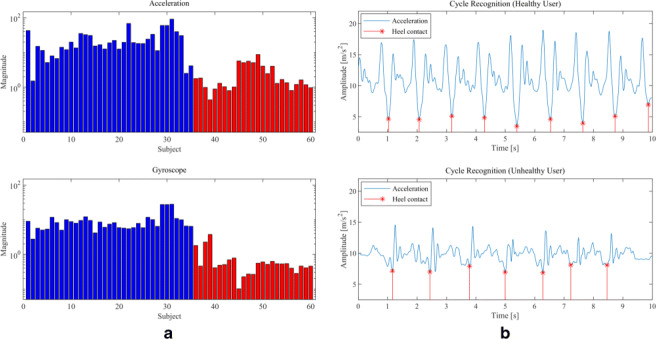
Graphical Abstract. This paper proposes a reliable monitoring scheme that can assist medical specialists in watching over the patient's condition. Although several technologies are traditionally used to acquire motion data of patients, the high costs as well as the large spaces they require make them difficult to be applied in a home context for rehabilitation. A reliable patient monitoring technique, which can automatically record and classify patient movements, is mandatory for a telemedicine protocol. In this paper, a comparison of several state-of-the-art machine learning classifiers is proposed, where stride data are collected and processed by using a smartphone(see figure). The main goal is to identify a robust methodology able to assure a suited classification of gait movements, in order to allow the monitoring of patients in time as well as to discriminate among a pathological and physiological gait. Additionally, the advantages of smartphones of being compact, cost-effective and relatively easy to operate make these devices particularly suited for home-based rehabilitation programs.

Graphical Abstract. This paper proposes a reliable monitoring scheme that can assist medical specialists in watching over the patient's condition. Although several technologies are traditionally used to acquire motion data of patients, the high costs as well as the large spaces they require make them difficult to be applied in a home context for rehabilitation. A reliable patient monitoring technique, which can automatically record and classify patient movements, is mandatory for a telemedicine protocol. In this paper, a comparison of several state-of-the-art machine learning classifiers is proposed, where stride data are collected and processed by using a smartphone(see figure). The main goal is to identify a robust methodology able to assure a suited classification of gait movements, in order to allow the monitoring of patients in time as well as to discriminate among a pathological and physiological gait. Additionally, the advantages of smartphones of being compact, cost-effective and relatively easy to operate make these devices particularly suited for home-based rehabilitation programs.

## Introduction

An important field of application for data classification and screening is the one concerning human motion, in particular the one required in gait analysis, defined as the systematic study of human walking [1–5]. As far as we know, considerable information can be extracted by analyzing patients’ walking because it contains important biometric features. In effect, gait is related to the walker’s physical and, sometimes, psychological state [6].

Decision-making for gait analysis could be supported by the use of computational intelligence techniques for automatic determining the status of a patient. For instance, in [7–9] several classification algorithms are evaluated and compared in terms of their ability to discriminate among physiological and pathological gait.

Generally, the most adopted tools for gait analysis are based on high complexity motion capture systems exploiting active or passive markers, electromyography (EMG), dynamometric platforms, and so on [10–14]. Unfortunately, the high cost and the complexity they require make them suitable only for specific clinicians and hospitals.

To ease the monitoring of patients’ motion, while keeping the cost low, a lot of researchers focus their attention on IMU sensors [15–17]. Unfortunately, these sensors have severe drift problems, making necessary their usage in parallel with other technologies [18]. To overcome these issues, some studies have analyzed the impact that new technologies based on both visual and non visual systems could bring on the related research and application fields [6, 19–21].

In effect, in the last decade, everyday life has been powerfully influenced by technology, like tablets or smartphones ceaselessly connected through mobile networks. Research is increasingly striving towards the exploitation and the evolution of daily experiences by improving and developing new functionality that people could ask for.

A current and quite recent trend of research has seen the investigation of smartphone-based applications for movement monitoring and analysis, as for example considered in [22]. Gait analysis experiments using a smartphone to demonstrate the capability to accurately quantify gait parameters with a sufficient level of consistency have been performed in [23] and in several other works as, for instance, in [24–27]. However, these approaches mainly rely on human experts (i.e., doctors) for the clinical analysis of smartphone data and making a decision accordingly. The feasibility, efficacy and usefulness of machine learning techniques for discriminating automatically the gait movements and for assessing the main extracted features have not been systematically evaluated so far.

In this work, we extend the use of sensors contained in a smartphone to realize a work particularly suited for a future home-based rehabilitation approach. In particular, its advantages of being compact, cost-effective and relatively easy to operate automatically, compared to the other onerous and expensive technologies, make this device particularly suited for this context. As for the biomedical context, a low-cost smartphone-based system could bring great advantages to both diseased people and clinicians, by upgrading the patients’ quality of life and reducing the average rehabilitation cost. Despite this approach being interesting for the treatment of many diseases like Parkinson’s, multiple sclerosis and Coxarthrosis, we will describe a specific solution tailored to the monitoring of people recovering from a stroke. In effect, post-stroke rehabilitation has been proven to be essential and effective in helping stroke patients to gradually regain part of their body functionality. In particular, gait analysis, which is the standard practice for diagnosis, assessment, monitoring and discussion of diseases that affect gait, is used to detect the walking patterns and posture that are unique for hemiplegic patients at different recovery stages.

We propose a reliable remote monitoring scheme that can assist medical specialist in watching over the patient’s condition. A smartphone is used to collect stride data and obtain useful information from these data by means of advanced features extraction methods. The system should be used to assist medical specialists in analyzing the rehabilitation path at range, also when the patient is not in the hospital anymore. In this way, assuming that the approach is inserted in a well-scheduled program of “home” rehabilitation, it will be possible to reduce costs, while improving the patient’s life quality and allowing clinicians to evaluate patient’s improvements in a safer and faster manner.

The novelty, with respect to state-of-the-art applications, is the combination of the data acquisition and filtering from the device, with data fusion and pattern recognition techniques that provide a correct definition of the gait movements, allowing to monitor the patient in time, as well as discriminate among a pathological and a physiological gait.

The rest of the paper is organized as follows. We introduce the proposed approach in Section 2. The application is ascertained by extensive computer simulations and several benchmark results, which are reported in Section 3 and discussed in Section 4. Finally, our conclusions are drawn in Section 5.

## Methods

### Selection and description of patients

In the experimental process, which will be described in the detail successively, we evaluate the gait of two set of individuals. A group of both healthy people and post-stroke patients took part in the experiment. We collected 60 different walking trials through heterogeneous smartphone devices of different manufacturers. Among these 60 records, 25 of them belong to voluntary unhealthy patients from the Rehabilitation Medical Center of the 2nd Hospital of Jiaxing, Zhejiang province, China; the remaining 35 are healthy persons among academic researchers and doctors of the previously cited Medical Center. Additionally, data differ for the length of the recording session: 41 of them (respectively 13 from patients and 28 from healthy people) are recorded in 10 s, the remaining 19 (12 of which are patients) are recorded in 20 s. People are asked to perform a walking in a straight path, without deviation.

All research activities in this study were conducted in accordance with the ethical principles of the Declaration of Helsinki. As it involves human participants, the present study was performed in accordance with the relevant institutional and national guidelines, with informed written consent from all human subjects involved in the study including for publication of the results. However, the study is exempt from the explicit ethics approval of appropriate institutional Committees, as it is mainly focused on the engineering aspects pertaining to the use of specific ICT technologies as well as signal processing and analysis of the related data. All subject anonymity is preserved as identifying information is not included in the manuscript.

### Procedural information

**Fig. 1:**
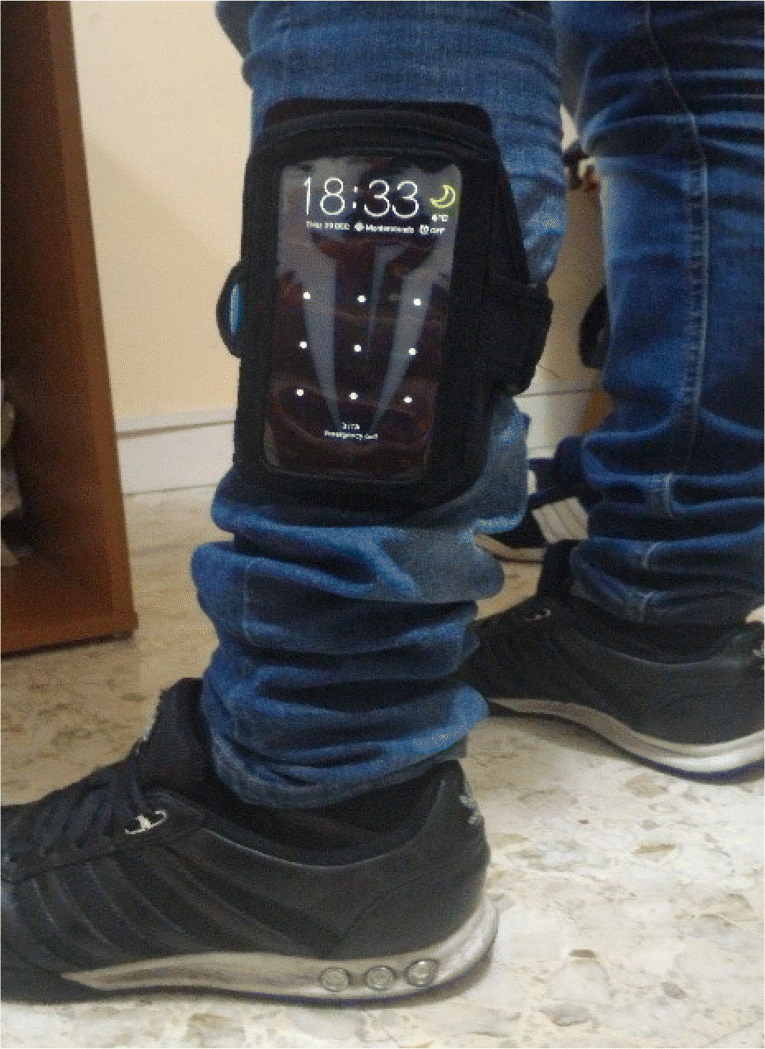
Position of the smartphone device during the clinical trials

In this study, we perform an analysis of the user’s stride in order to extract suitable features for a classification purpose. A low-cost smartphone device (i.e., Samsung’s Galaxy A3 2017 SM-A320F with Android v6.0.1) is put in the pocket of a band and ties around the user’s calf as shown in Fig. 1. In fact, most of the gait information and lower limb’s angle movements are linked to this muscle. One device only is adopted, so as to represent a realistic scenario in a home-based context where a user has one smartphone only.

**Fig. 2:**
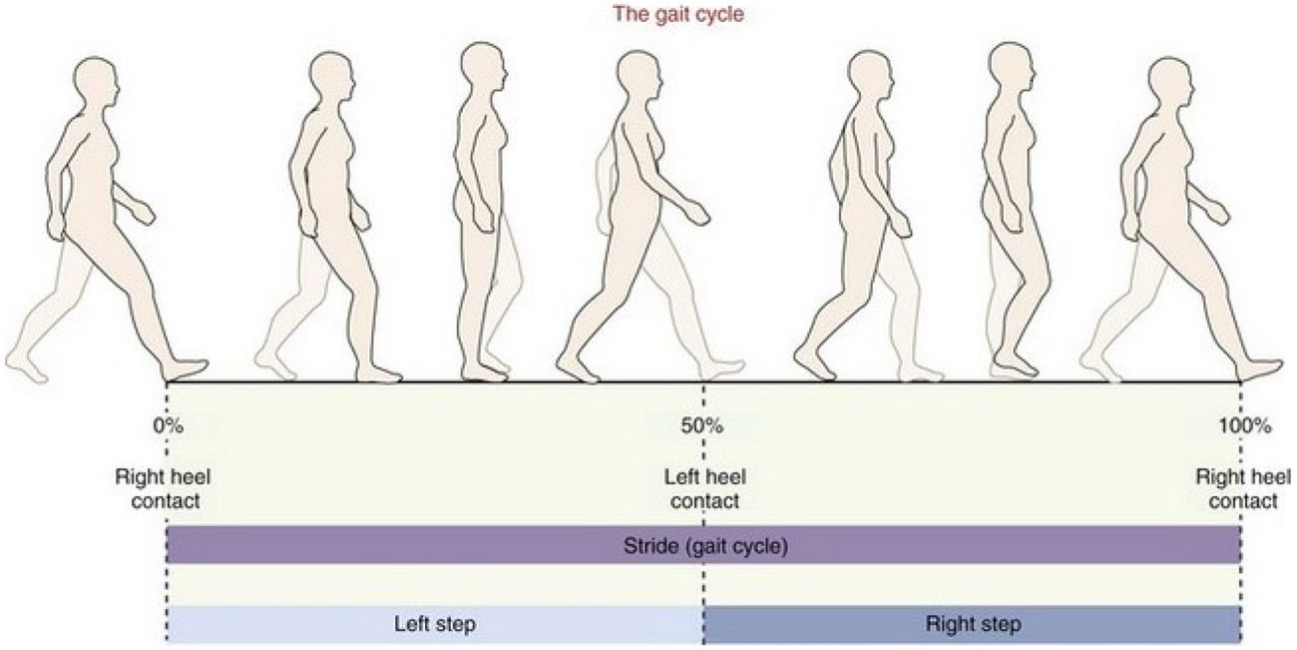
The general gait cycle

In order to implement a simple and cheap approach, we decided to use one smartphone only in the clinical trials. Further researches might investigate on the use of two or more devices, although dealing with severe issues as time synchronization and sensor mismatch among smartphones. Consequently, we are not able to provide a full representation of the gait cycle, which is represented in Fig. 2, since one sensor node is not able to achieve this. Rather, our aim is to evaluate some additional features that could be used for a possible home-based lower limb rehabilitation, by focusing on the features in Table 1 that should be easy to recover using the adopted hardware setup.

**Table 1:** Spatio-temporal features adopted for the gait analysis

Feature	Unit	Description
Mean cycle duration	s	Mean value of the stride duration during a trial
Cycle regularity	s	Standard deviation of the stride duration during a trial
Cadence	step/min	Number of strides executed in a given time
PSD peak acceleration	dB	Maximum value of the acceleration’s PSD
PSD peak gyroscope	dB	Maximum value of the gyroscope’s PSD

**Fig. 3:**
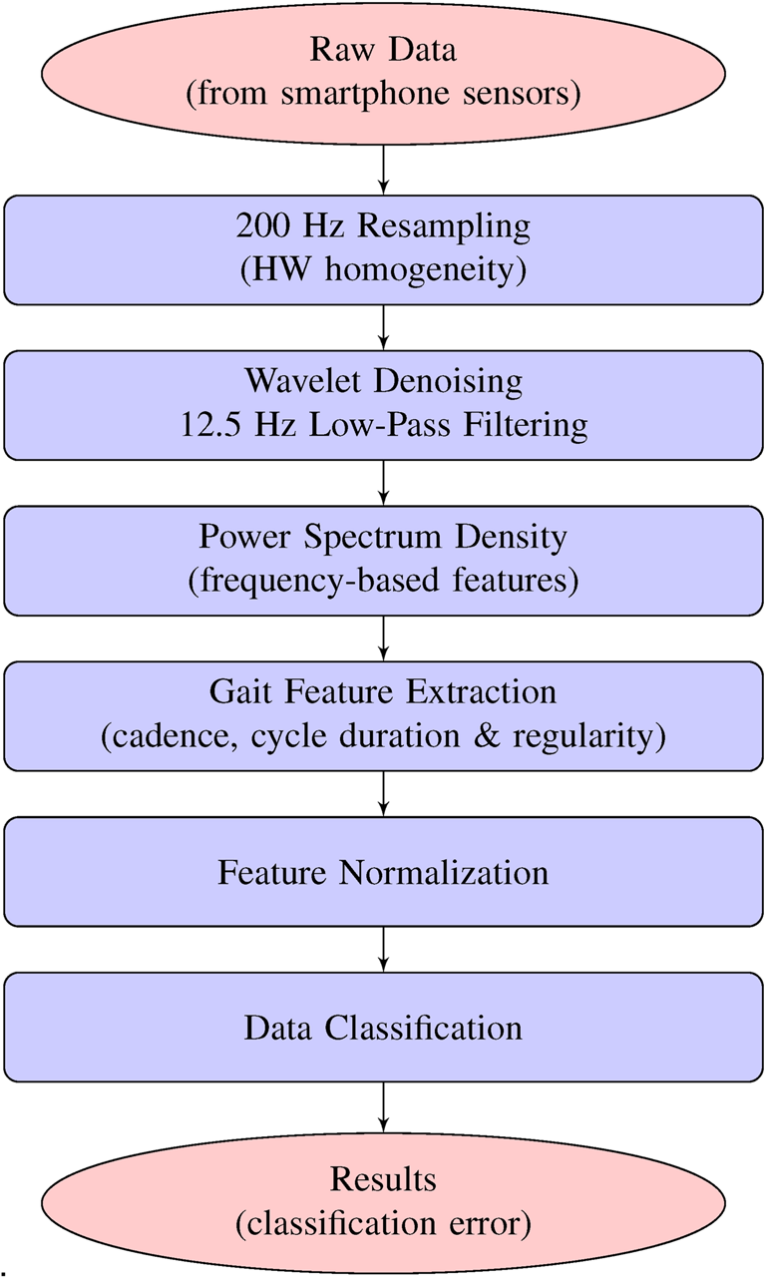
Flow chart of the proposed processing system

The proposed system for gait monitoring is illustrated in the flow chart of Fig. 3 and it can be summarized into several main operations, described in the following subsections.

#### Raw data collection from sensors

Firstly, data acquisition is performed as follows:

- the user opens the application (we worked in this case on an Android^®;^ device but any operating system can be adopted) and sets the recording time;
- the smartphone is put into the band’s pocket and fasten around the calf;
- the user taps the “Start” command on the screen and, after a countdown of 3 s, the app begins to record data;
- during the recording time, the user performs the walking test and a device’s vibration will advise him/her that recording is terminated;
- at the end of the trial, the user uploads data into the database after explicit consensus granted via the application.

If the user is a voluntary hemiplegic patient, an assistant helps him/her in any demands. Accelerometer, gyroscope, and magnetometer data are captured during each trial. However, in the following we will not consider magnetometer data because they are too sensitive to the presence of metal objects in the environment.

#### Data resampling

Once data are collected from sensors, the second main step consists in resampling them in order to reduce the differences caused by the fact that acquisition is carried out using different smartphones with heterogeneous control hardware and sensor technologies. In addition, the app code usually cannot set the sampling rate at software level, as it depends on the hardware available on the adopted device. Depending on which device is used, each sensor data is sampled at a different rate (i.e., from 50 to 350 Hz). In order to apply the same denoising algorithm to the whole set of data, we need a common sampling rate. Since 200 Hz is a reasonable trade-off, also considering the final target rate after wavelet filtering discussed successively, depending on the starting rate we did resampling or low-pass filtering to obtain the same rate.

#### Denoising and filtering

The successive step consists in denoising both acceleration and gyroscope data by a wavelet-based estimation algorithm and, successively, in low-pass filtering the reconstructed signals in order to make easier the feature extraction process.

A wavelet-based denoising algorithm is firstly applied by using the following model [28]:

1 $$ s(n)=f(n)+\sigma e(n) , $$

where *n* is the sample (time) index, *s*(*n*) is the noisy signal, *f*(*n*) is the signal to be recovered, *e*(*n*) is a zero-mean, unit-variance Gaussian white noise and *σ* is the noise level. The adopted algorithm is able to suppress the noise part of the signal *s*(*n*) and to recover *f*(*n*) through the following steps:

1. a wavelet decomposition of *s*(*n*) at level *W* is evaluated (we used the family of Daubechies’ least asymmetric wavelets as default option);
2. a thresholding operation is performed to detail coefficients for each computed level from 1 to *W*;
3. the wavelet reconstruction is computed based on the level *W* original approximation coefficients and on the modified detail coefficients from level 1 to *W*.

In the following, we will consider *W* = 4 levels taking into account a minimum of 128 samples per data recording trial. Noise estimate is performed at each wavelet level to scale the reference noise model *σ* = 1, then a soft thresholding is performed by using a “universal threshold” approach for minimax performance [29], using a threshold proportional to $\sqrt {2\ln (L)}$ where *L* is the length of the considered signal.

**Fig. 4:**
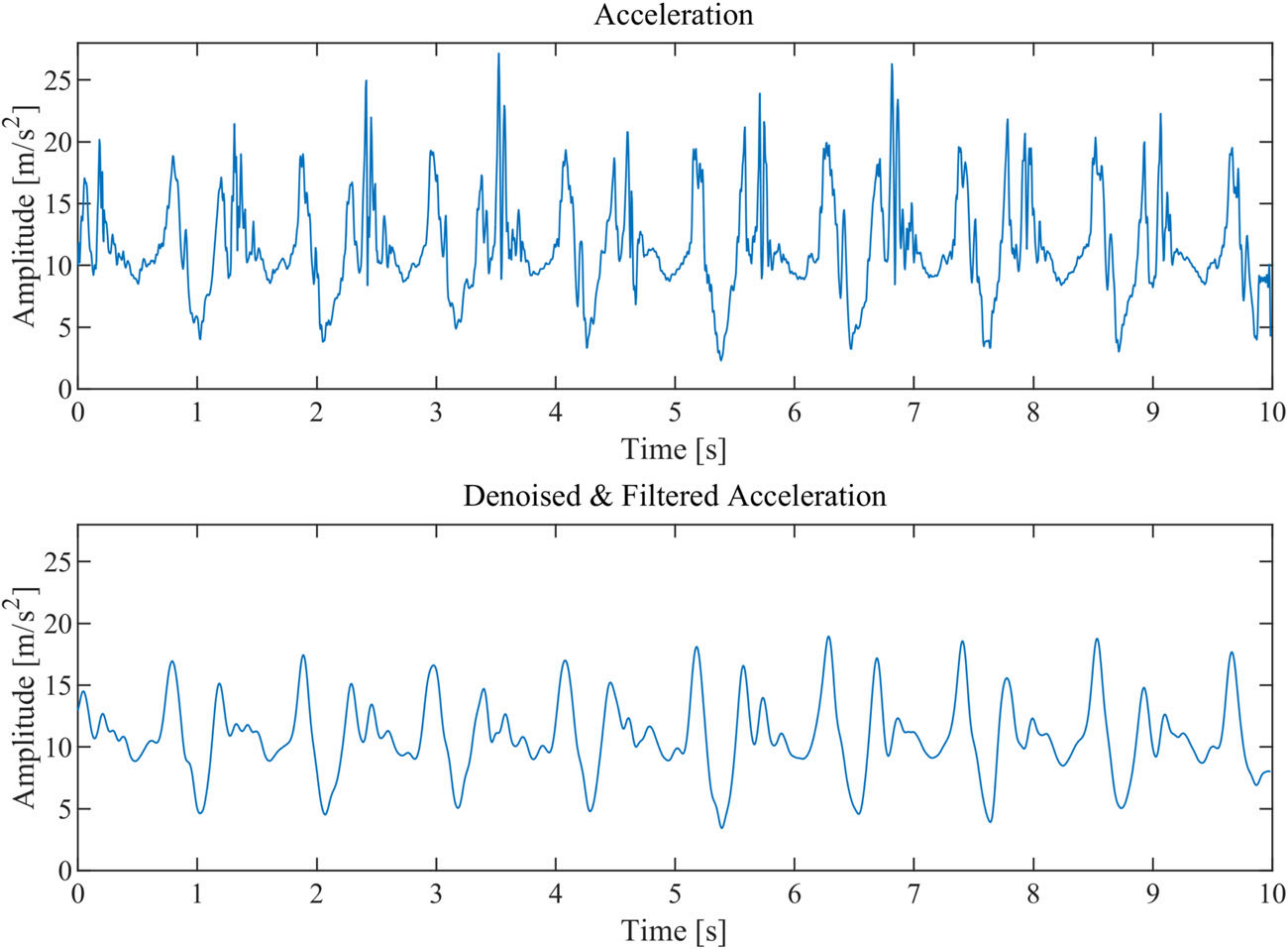
An example of denoising and filtering on the absolute value of the acceleration signal

After denoising, acceleration and gyroscope signals are passed through a 4-level Mallat’s filter bank [30] for low-pass filtering and downsampling. In fact, average walking frequency of healthy people is about 1.8 Hz [31] and hence, as shown in Fig. 4, a final sampling frequency of 12.5 Hz is suitable to make easier the subsequent feature extraction process without loosing any useful information.

#### Power spectrum density estimation

**Fig. 5:**
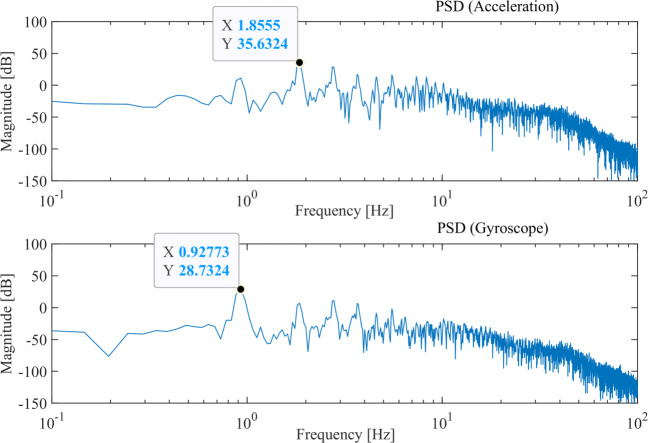
An example of PSD extraction and peak detection on denoised acceleration and gyroscope data at the original sampling rate of 200 Hz

As a well-known result achieved in the literature [32], the feature extraction for gait classification analysis should be based also on the power spectrum density (PSD) of considered signals, which coincide with the absolute values of acceleration and gyroscope signals after denoising and filtering. PSD estimation is then performed by the “Periodogram” method and we used the maximum PSD magnitude values (i.e., peaks) for both acceleration and gyroscope data. An example in this regard is shown in Fig. 5, where input denoised unfiltered data are shown.

It is worth to point out that the average stride frequency of every stride can be estimated and used as a parameter to find the walking cycle in each trial; in most cases, the acceleration’s average frequency (PSD peak) is located at the gyroscope’s second harmonic (see Fig. 5). However, because of relevant fluctuations due to the mechanism through which data are measured, this rule could not be always satisfied.

#### Gait feature extraction

In addition to the PSD peaks introduced before, other three features are used for gait cycles discrimination: cycle duration (*C*_*d*_), cycle regularity (*C*_*r*_), and cadence or revolutions per minute (*R*_*m*_).

Those features are generally used by the clinicians, in combination with other information on the subjects, to monitor the progress of a therapy or the evolution of a disease [5, 33, 34]. By the proposed approach, we propose a novel use of these features through the synergy of extracting gait features by low-cost devices and making classification automatically by means of machine learning models.

**Fig. 6:**
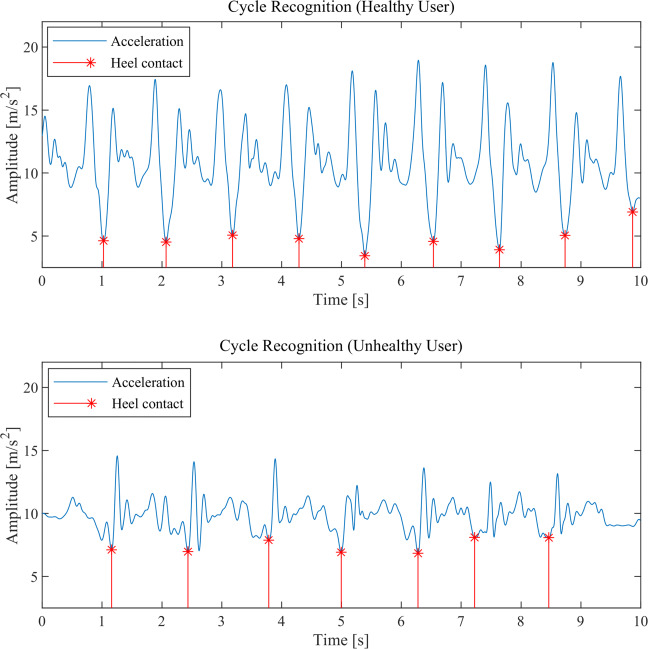
An example of stride recognition using the absolute value of the time acceleration pattern for a healthy and unhealthy user

Each stride is recognizable from the acceleration pattern as the time between two “valleys”. In fact, when the foot hits the ground, the sudden acceleration causes a spike followed by a deceleration that is represented by a valley; then, the successive leg swinging causes a new acceleration and the process is repeated cyclically. However, this behavior is more evident in healthy people rather than hemiplegic ones, as shown in Fig. 6.

In order to find out the time instants of foot contacts, both accelerometer and gyroscope data are considered. First, the absolute PSD’s peak of both accelerometer and gyroscope data is measured; from the related frequency we can obtain a gross estimate of the average time between two steps considering either acceleration or gyroscope data, respectively. The average of these time intervals is then used as a rolling window to find a minimum in a small interval around an initial guess in accelerometer data, the final result is shown in Fig. 6.

Since we assume that the first valley is the starting point of the first stride and that one stride is performed during the time gap between two consecutive valleys, we can define the cycle (stride) duration as:

2 $$ C_{d}[k]=V[k+1]-V[k] , $$

where *V* [*k*] is the array containing the valleys’ locations (in seconds) and *k* is their index. So, *C*_*d*_[*k*] will represent the time difference between two valleys of a gait cycle. The cycle (stride) regularity is expressed as the standard deviation of the elements in the vector *C*_*d*_, it is measured in second and proves regularity when the value of *C*_*r*_ tends to zero. Finally, the cadence is defined as:

3 $$ R_{m} =\frac{60 N_{c}}{V_{l}-V_{f}} , $$

where *N*_*c*_ is the number of cycles taken from recorded array, *V*_*l*_ is the last element of the valley location (in seconds) and *V*_*f*_ is the first one. Consequently, the cadence is the projection of how many strides could be performed in a minute and it is thus expressed in cycles/min.

All of the features adopted in this paper are summarized in Table 1; for the cycle duration representing a recorded trial we consider the average value of the elements in the vector *C*_*d*_.

#### Feature normalization

Before using data for classification purposes it is helpful to perform data normalization in order to scale the features in the same numerical range, which in this case is chosen between 0 and 1. Let *M* be the number of patterns in the available dataset, where each pattern **x**_*m*_, $m=1{\dots } M$, is a collection of *N* features (i.e., *N* = 5 in the present approach) associated with a specific recorded trial:

4 $$ \mathbf{x}_{m}=[x_{m_{1}}  x_{m_{2}}  {\dots}  x_{m_{N}} ] , m=1{\dots} M . $$

Since data features are completely heterogeneous, patterns cannot be normalized globally but with different affine transformations of features independent from one another:

5 $$ x_{m_{j}}\gets \frac{x_{m_{j}}-b_{j}}{a_{j}-b_{j}} , j=1 {\dots} N , m=1 {\dots} M , $$

where the terms are defined as ${a_{j}=\max \limits _{m}\{x_{m_{j}}\}}$ and ${b_{j}=\min \limits _{m}\{x_{m_{j}}\}}$, with ${j=1 {\dots } N}$.

#### Data classification and results

The last step of the proposed algorithm consists in training a binary classifier by using well-known machine learning paradigms in order to categorize data and discriminate between healthy and unhealthy people. This is useful also to understand if the considered gait features are able to support this kind of classification, as for many other application fields [35–38].

We have investigated in our experiments all of the possible combinations of input features, therefore considering 2^5^ − 1 = 31 different datasets. A 10-fold stratified validation is performed and several classification algorithms are compared in terms of classification error for each dataset.

### Statistics

Several classification algorithms are used to assess the validity of the proposed approach:

- Linear Discriminant Analysis (LDA): tries to characterize data using a linear polynomial in order to separate patterns into two or more classes. It maximizes the inter-class discriminatory information by using the Fisher Discriminant technique for surface separation [39, 40]. For the method to perform well data should satisfy the homoscedastic hypothesis, no hyperparameters are to be set in advance.
- Quadratic Discriminant Analysis (QDA): similarly to the LDA, tries to characterize a dataset using a quadratic polynomial based on Gaussian density conditional functions [41, 42]. It does not require any assumption on data, so it is more suitable for real contexts, no hyperparameters are to be set in advance.
- K-Nearest Neighbor (KNN): classifies a pattern depending on the most frequent class in the neighborhood of the pattern itself [43]. It does not require any assumptions on data and, in the following, we will use the Euclidean distance between patterns and *K* = 5 as a default value.
- Naive Bayes (NB): is a statistical technique that seeks to verify if an element belongs to a class based on Bayes’ Theorem [44, 45]. The algorithm calculates various conditional probabilities and assigns the patterns to the class with the highest probability. In the following we will use Gaussian kernel smoothing to estimate and model the data density.
- Support Vector Machine (SVM): is a particular supervised learning approach that can be applied for both regression and classification problems [46, 47]. Based on the solution of a quadratic convex problem, it is used for finding global minimum also in nonlinear complex problems. In the following we will adopt as default options a Radial Basis Function (RBF) kernel with Sequential Minimal Optimization (SMO) solver.
- Neuro-Fuzzy classifier (NF): is used to partition the feature space into fuzzy sets and assign non mutually exclusive membership values representing the reliability of the pattern of belonging to each class [48, 49]. In the following the model will be trained by a scaled conjugate gradient method with 100 maximum epochs and one cluster per class.
- Classification and Regression Tree (CART): operates by recursively splitting data until ending points, defined by some predefined criteria, are achieved [50, 51]. It should handle with nonlinear relations between features and classes [52], finding a correct trade-off among computational complexity and accuracy. Prior class probabilities will be estimated in the following based on class frequencies.
- Probabilistic Neural Network (PNN): this approach is based on a four-layer neural network employing Bayesian decision-making theory and data-driven learning [53, 54]. The spread of radial basis functions will be set by default to 0.1.
- Fuzzy Inference System (FIS): this method adopts first-order fuzzy rules and a data-driven inference system trained by means of the Substractive Clustering method [48, 55]. Gaussian membership functions will be adopted with one rule per fuzzy cluster and 0.5 influence of the cluster center (normalized data space).

All the classifiers use the same set of data, no ad hoc changes are made to make every dataset suited for the specific classification model. It can be underlined, however, that some general differences exist in the way by which each algorithm extract information from the data. For instance, KNN and SVM classifiers do not provide from training data a mathematical model of the classifier. In fact, SVM classifier gives as output the support vectors, while KNN seeks, for each pattern to be classified, the nearest patterns which the output label is extracted from.

On the contrary, statistical and fuzzy logic-based classification algorithms aim at finding, by using training data, the parameters of a mathematical model that is able to infer the probability of or the fuzzy membership to a class, respectively, for the pattern under classification. An intermediate behavior is the one of CART classifiers, where a decision tree is obtained by training data rather than a parametric model. Further details can be found in the references cited for each classifier listed above.

## Results

**Fig. 7:**
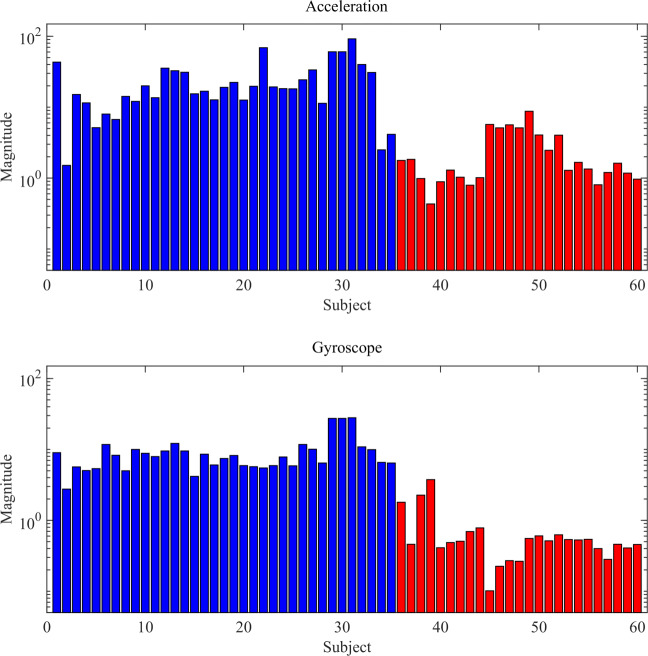
Maximum PSD magnitude of acceleration and gyroscope of healthy (blue) and unhealthy (red) subjects

In this section we report the obtained numerical results. For the sake of illustration, let us consider firstly the PSD magnitude of acceleration and gyroscope for healthy classification. Looking at Fig. 7, where the first 35 (blue) records are from the healthy group while the successive 25 (red) records are of the post-stroke patients, by the differences of the maximum PSD magnitude the reader could have a sufficient but not so accurate estimation of the healthy status. Consequently, a more accurate classification approach is required in order to perform a robust analysis.

To this end, we considered the classification models introduced in Section 2.3 and we performed an exhaustive search considering all the 31 sets of possible combinations of the 5 features listed in Table 1. A stratified 10-fold validation procedure was adopted for evaluating the classification accuracy; for each subset of features a classifier is trained 6 times, by classifying each time 10 different patterns (i.e., subjects or recorded trials) and using the remaining 50 patterns for training. As most of the classification models depend upon a random initialization of model parameters, and the 10-fold partitioning of the dataset is random as well, we repeated the above procedure 10 times and the ones considered in the following are the average values of accuracy obtained over the 10 different trials.

For each classifier we report in Table 2 the subset of features that yielded the best (average) classification accuracy, among all of the possible combination of features. More precisely, there are 5 rows (one per feature) and 10 columns (one per each algorithm plus the number of times that each feature is selected in an optimal dataset); each element of the table takes value 1 if the corresponding feature is selected and 0 otherwise. In case of identical values we chose the subset with the lower number of features according to a regularization approach [56].

**Table 2:** Best feature subset per classifier and number of times a feature is adopted

Feature	LDA	QDA	KNN	NB	SVM	NF	CART	PNN	FIS	Occurrences
Mean cycle duration	0	1	1	0	0	1	0	0	0	3
Cycle regularity	0	0	0	1	0	0	0	0	0	1
Cadence	0	1	0	0	1	0	0	1	0	3
PSD peak acceleration	0	0	1	0	0	0	0	0	0	1
PSD peak gyroscope	1	1	1	1	1	1	1	1	1	9

The numerical classification results are summarized in Table 3 where, for each classification algorithm, there are reported the average classification accuracy and the related standard deviation over the 10 trials carried out in correspondence of the subset of features that yielded the best (average) accuracy. The number of adopted features is obtained by summing the ones in the related column of Table 2.

**Table 3:** Average classification accuracy and standard deviation for the best feature subset

Classifier	Average accuracy (%)	Std. deviation (%)	Adopted features
LDA	83.23	± 1.28	1
QDA	88.39	± 1.35	3
KNN	87.47	± 1.25	3
NB	90.38	± 0.89	2
SVM	89.84	± 1.38	2
NF	89.41	± 1.12	2
CART	87.74	± 1.07	1
PNN	91.13	± 1.12	2
FIS	86.40	± 1.19	1

## Discussion

By analyzing the results obtained in Table 2, it is evident that the acceleration is not a useful feature while the gyroscope must be taken into account for a good discrimination. In fact, the sole use of the PSD Peak Gyroscope feature is the best option for LDA, CART, and FIS classifiers. On the other hand, QDA, SVM, and PNN are able to achieve a good classification by using some other features, such as Cadence, which is sufficient for SVM and PNN. It is worth to point out that these results are quite in accordance with current medical practices.

Looking at the overall performance of the proposed classification approaches, we note that the accuracy in discriminating among pathological and physiological gait is always maintained at high levels, from 80 to 90%. In addition to PSD peak gyroscope, cadence is the feature that by means of PNN is able to obtain the best accuracy of 91.13%. Cycle regularity allows NB classifier to achieve a 90.38%, which the second score in the ranking. In all cases, the performance volatility measured by the standard deviation is adequate. Overall, the great performance of PNN with only 2 features does suggest that a data-driven machine learning approach can bring improvements with respect to statistical approaches based, for instance, on Discriminant Analysis and to non-parametric models as KNN as well.

As a final remark, we note that the previous numerical results are strictly dependent on the uncertainty of measures through which data are gathered and then processed. In the present case, error in measurements depends by two main factors: accuracy and precision of hardware sensors; objectivity of the experimental setup, mainly depending on the application of the smartphone on a same point of the body as well as on the reproducibility of clinical trials (same walking, same movements, etc.). In this work, the influence of such errors is mitigated by the use of several and different hardware devices and by the adoption of a relatively large number of patients during the clinical tests.

## Conclusions

The novelty with respect to state-of-the-art applications is the combination of data acquisition and filtering on the device, with pattern recognition and data fusion techniques that provide a correct discrimination of gait movements. An exhaustive feature selection approach is considered in order to find out the best subset of features able to discriminate among healthy and unhealthy subjects.

The procedure has been used also for evaluating the performance of several classification models in terms of classification accuracy. Very good performances, even achieving a 100% of accuracy, are obtained on the clinical trials performed in this research. It is important to point out that this is a feasibility study, not a clinical trial of a model. However, the results are very promising for making possible to assist medical specialists in analyzing the rehabilitation path in the near future.

In particular, the model could be extended for using it in specific and personalized programs for home rehabilitation meant to improve the patient’s quality of the life while boosting the treatment effectiveness and thus shortening the patient’s recovery time.
